# Traumatic Macular Hole: Clinical Management and Optical Coherence Tomography Features

**DOI:** 10.1155/2020/4819468

**Published:** 2020-10-20

**Authors:** Chunling Lei, Li Chen

**Affiliations:** ^1^Department of Ophthalmology, Xi'an No. 4 Hospital, Xi'an, Shannxi Province, China; ^2^Department of Ophthalmology, Beijing ChaoYang Hospital, Capital Medical University, Beijing, China

## Abstract

The complex and uncertain prognosis of traumatic macular hole (TMH) makes it a difficult and challenging problem in clinical management. The features of spontaneously closed TMH and the time of vitrectomy remain unclear. This retrospective study aimed to demonstrate the optical coherence tomography (OCT) features of TMH, explore the relationship between OCT parameters and visual outcomes, and further evaluate the therapeutic effect of surgical management. Seventeen TMH patients were included in this study. 13 eyes of TMH received vitrectomy surgery and 4 eyes of TMH were closed spontaneously. Baseline patient characteristics, surgical details, and 6-month postoperative follow-up clinical assessment were recorded prospectively. There was a moderate rate (4/17 eyes, 23.5%) of spontaneous closure. The mean time of hole closure was 9.5 ± 9.9 weeks, and 75% occurred within three months. In the spontaneously closed TMH eyes (*n* = 4), an intact ellipsoid band was observed in all four patients with a mean age of 12.0 ± 1.6 years and a smaller preoperative basal diameter of 418.0 ± 283.6 *μ*m. Small basal diameter of the macular hole at baseline (*p* = 0.02) was associated with spontaneous closure of TMH acuity. In the vitrectomy surgery group (*n* = 13), an intact ellipsoid band was observed in four patients (4/13) with a mean age of 27.0 ± 12.7 years and a larger preoperative basal diameter of 943.0 ± 444.2 *μ*m (*p* = 0.02). Vitrectomy results in a better closure rate (11/13 eyes, 84.6%). Combined with the spontaneously closed TMH eyes, the overall hole closure rate was 88.2% (15/17 eyes). After 6-month treatment for all patients, the best-corrected visual acuity (BCVA) increased to 0.59 ± 0.40 (logMAR) compared to baseline 1.01 ± 0.50 (logMAR) (*p* < 0.001). The ellipsoid band integrity was found to be closely correlated with visual acuity (*p* = 0.03). In conclusion, vitrectomy is an effective treatment for TMH. Surgical management for TMH achieved better anatomical closure and improved visual outcomes. Observation for 3 months may be considered before deciding if surgical intervention is suitable.

## 1. Introduction

Macular holes are full-thickness defects of the neuroretina that disrupt the foveal contour. They are commonly idiopathic or age-related but may be traumatic due to blunt injury to the eye. The first case of traumatic macular hole (TMH) was described by Knapp [1] in 1869. The incidence of TMH is 1.4% in closed-globe trauma and 0.15% in open-globe injuries [2]. Due to its rarity, studies on TMH are limited [3].

Idiopathic macular hole (IMH) occurs more generally among women over 65 years of age. However, TMH occurs most often in young people and is caused by a sudden extrinsic force, which creates dynamic forces within the sclera and vitreous. The forces lead to a wide range of retinal pathologies, including commotio retinae, diffuse retinal edema, retinal hemorrhage, retinal tears, vitreous hemorrhage, choroidal rupture, and photoreceptor and retinal pigment epithelium (RPE) damage [4]. All these pathological changes will eventually result in severe vision loss. Due to the low incidence of TMH, currently, there are no standard clinical guidelines for the clinical characteristics, treatment, and prognosis of TMH.

According to the follow-up reports on TMH cases, the rate of spontaneous closure of TMH is reported to range from 10% to 67% [5–9]. A meta-analysis of surgical outcomes of vitrectomy for TMH revealed a successful closure rate of 83% [1]. In the observation of TMH prognosis and follow-up, there have been relatively few studies on the correlation between optical coherence tomography (OCT) and prognosis of TMH. Herein, we present a follow-up case series of consecutive patients with TMH. By retrospection of both initial and follow-up OCT data, we analyzed the clinical and OCT characteristics and effects of operation in detail in order to provide valuable information regarding the treatment and prognosis of TMH.

## 2. Materials and Methods

This is a retrospective case series of 17 patients diagnosed with TMH who were evaluated at our hospital outpatient clinic from 2014 to January 2017. The study was approved by the Ethics Committee of the No. 4 Xi'an Hospital. The study adhered to the tenets of the Declaration of Helsinki.

Baseline assessment included age, sex, laterality, nature of blunt ocular trauma, and time lapse between trauma and first presentation. Baseline best-corrected visual acuity (BCVA) was recorded in decimal notation and converted to a logMAR value for statistical analysis. All cases were subjected to detailed ophthalmic examination, including BCVA, slit lamp of the anterior segment, OCT examination, fundus slit-lamp biomicroscopy, and color fundus photography. These cases were followed up for at least 6 months.

### 2.1. Inclusion and Exclusion Criteria

Inclusion criteria were a clear traumatic history with diagnosis of full-thickness MH, which was confirmed by indirect ophthalmoscopy, color fundus imaging, and OCT. Exclusion criteria were patients with any other eye diseases that affect vision and prognosis, or with open-globe injuries, severe cataract or vitreous hemorrhage, and retinal detachment. Those patients who had received laser or surgical treatment before or who could not be reached for regular follow-up also were excluded.

### 2.2. Spectral Domain OCT Examination

**Figure 1:**
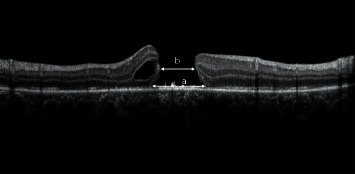
The hole basal diameter (a) was measured as the hole diameter at the level of the retinal pigment epithelium. The hole minimum diameter (b) was measured as the minimum inner diameter of the hole.

Spectral domain (SD) OCT is the key technique in the management of TMH. It allows for a detailed assessment of the macular hole parameters, vitreoretinal interface, and other associated macular changes at each presentation. In our study, OCT scans were performed using one of the two SD-OCT versions: the Topcon 3D OCT-2000 (Topcon, Tokyo, Japan) or the Heidelberg Spectralis (Heidelberg Engineering, Inc., Heidelberg, Germany). Several SD-OCT parameter measurements were recorded, including height (internal limiting membrane (ILM) to RPE) and the minimum and basal diameter of the hole (Figure 1).

During the follow-up, if the OCT examination revealed a persistent open hole for three months or increased basal diameter, observation was stopped and vitrectomy was performed. For patients presenting signs of hole basal diameter decreasing, OCT was reexamined weekly to observe hole size, especially in the first month. During subsequent follow-up, OCT was reexamined monthly or every 2 months. Finally, observation was stopped once the hole was completely closed.

### 2.3. Surgical Method

The surgical procedure was explained to all patients or their relatives, and written consent was obtained. A standard 23 G three-port pars plana vitrectomy (PPV) was conducted, followed by induction of posterior vitreous detachment if not already detached. After ILM staining with indocyanine green dye, the ILM around the hole was peeled at least 2 disc diameter areas in order to release the traction. ILM was not completely lifted off the retinal surface; rather, peeling was stopped at the edges of the hole, creating a free-floating flap that was left attached all around the edges of the hole. Then, fluid-air exchange was conducted followed by injection of air, perfluoropropane (C3F8), or hexafluoroethane (C2F6). Based on the literature review, [10] we improved the surgical technique by using autologous serum to promote the healing of the hole in three patients with a large macular hole. Sclerotomy ports were removed, and their sites were tested for any leakage. Postoperatively, combined antibiotic-steroid drops (Tobradex eye drops, Alcon, Fort Worth, USA) were used four times daily for 4 weeks. For gas-filled eyes, the patients were instructed to maintain a face-down position whenever possible for 1 week or until 50% of the gas bubble was absorbed.

### 2.4. Pattern of Hole Closure

According to previous literature studies [11, 12], there are different types of hole closure for postoperative anatomical outcome of IMH and TMH. In Grade A, the macular hole is closed with a bridge-like closure. In Grade B, the macular hole is completely closed with a foveal morphology appearance. In Grade C, the macular hole is poorly closed, with an absence of foveal-area neurosensory retina. For unclosed macular holes, the edge of the macular hole was attached to the RPE.

### 2.5. Postoperative Follow-Up

After operation, follow-up occurred at postoperative day 1, week 1, month 1, month 3, and month 6. BCVA was recorded at each visit. The follow-up period, pattern of TMH closure, and grade of ellipsoid zone recovery were observed using SD-OCT, especially for the outer retinal layers, including the external limiting membrane and the inner segment/outer segment photoreceptor junction layers.

### 2.6. Statistical Analysis

Comparisons of means were analyzed using the unpaired Mann–Whitney *U* test or paired *t*-test. All statistical tests were two-tailed, and significance was defined as *p* < 0.05. GraphPad Prism 8.0 (GraphPad Software, California, USA) was used for all statistical analyses.

## 3. Results

### 3.1. General Data

**Figure 2:**
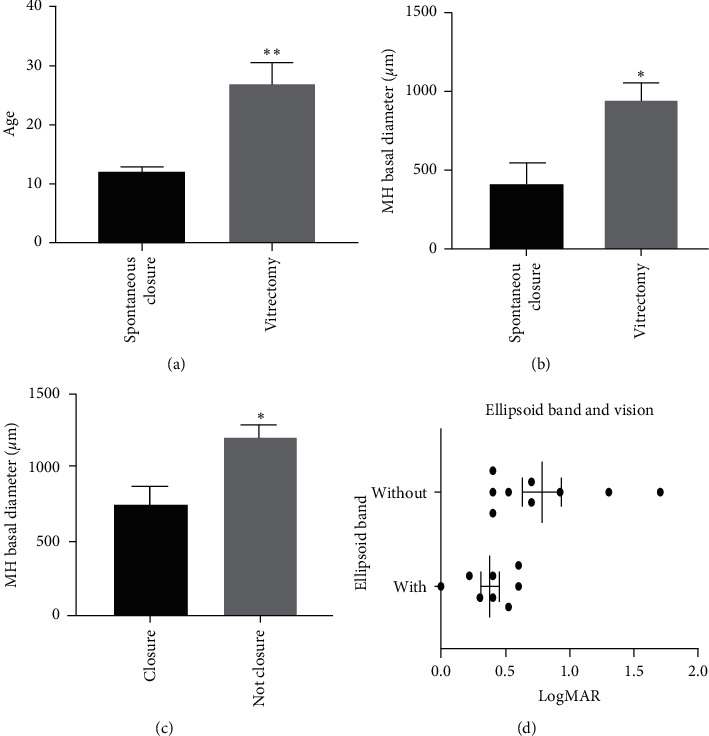
(a) Patients' mean ages for both groups. The mean age was 12.0 ± 1.63 years in the spontaneous closure group and 27.0 ± 12.7 years in the vitrectomy group (*p* = 0.009). (b) In the spontaneous closure group, the basal diameter was 418.0 ± 283.6 *μ*m, whereas the diameter was 943.0 ± 444.2 *μ*m in the vitrectomy group (*p* = 0.02). (c) The preoperative basal diameter was 754.8 ± 475.4 *μ*m in the eyes with successful macular hole closure and 1204.5 ± 132.2 *μ*m in the eyes without macular hole closure (*p* = 0.02). (d) The relationship between ellipsoidal band and postoperative BCVA. The eight cases with ellipsoid band integrity demonstrated better average BCVA than those without ellipsoid band integrity (*p* = 0.03).

Seventeen patients (17 eyes) with TMH were included during the study period, including 15 males and 2 females. The age of TMH patients ranged from 12 to 45 years (mean: 23.5 ± 12.5 years). All cases were caused by incidents of closed-globe trauma, including boxing, ball sports, firecrackers, and laser pen injury (Table 1). Among these 17 patients, 13 cases (76.5%) received vitrectomy, while 4 cases (23.5%) demonstrated spontaneous closure. The mean age of the spontaneous closure group was 12.0 ± 1.6 years (range: 10–14 years), whereas the mean age the vitrectomy group was 27.0 ± 12.7 years (range: 12–45 years) (*p* = 0.009) (Figure 2(a)).

### 3.2. Hole Closure

Four (23.5%) of the TMHs closed spontaneously, and the meantime of hole closure was 9.5 ± 9.9 weeks (range: 2–24 weeks). Two of these spontaneous closures occurred between 2 and 4 weeks, one occurred at 8 weeks, and one occurred more than 24 weeks after presentation. Thirteen patients underwent vitrectomy; of these, 11 patients (84.6%) had successful closure. The median time from trauma to intervention was 2.5 ± 0.8 months. Patients presented with three grades of anatomical outcomes: one case was Grade A, seven cases were Grade B, and five cases were Grade C. Two cases of TMH were not closed. OCT shows the absence of foveal-area neurosensory retina, but the edge of the macular hole was attached to the RPE.

### 3.3. OCT Characteristics at Presentation

In OCT examination, preexisting posterior vitreous detachment (PVD) and intraretinal cysts have a relation with TMH formation. In our study, two of the 17 eyes were found to have PVD. Intraretinal cysts were found in one of the four patients (25%) in the spontaneous closure group and four of the eleven patients (36.6%) in the vitrectomy group.

During the follow-up, OCT showed a significantly smaller basal diameter of 418.0 ± 283.6 *μ*m in the spontaneous closure group compared with 943.0 ± 444.2 *μ*m in the vitrectomy group (*p* = 0.02) (Figure 2(b)). Additionally, in the spontaneous closure group, the smaller minimum diameter and height of hiatus were 220.0 ± 129.3 *μ*m and 424.0 ± 237.1 *μ*m, respectively, and in the vitrectomy group, the smaller minimum diameter and height of hiatus were 520.0 ± 129.3 *μ*m and 471.0 ± 218.3 *μ*m, respectively (*p* = 0.05). The basal diameter of patients with hole closure is significantly smaller than that of not closed (754.8 ± 475.4 *μ*m vs. 1204.5 ± 132.2 *μ*m, *p* = 0.02) (Figure 2(c)).

The ellipsoid band integrity was found to be closely correlated with visual acuity. In our study, the average BCVA was logMAR 0.38 ± 0.20 in the eight cases that retained ellipsoid band integrity. However, in the nine patients with incomplete ellipsoidal bands, an average BCVA was logMAR 0.78 ± 0.48. Greater ellipsoid band attenuation was found in eyes with worse postoperative visual acuity (*p* = 0.03) (Figure 2(d)).

**Figure 3:**
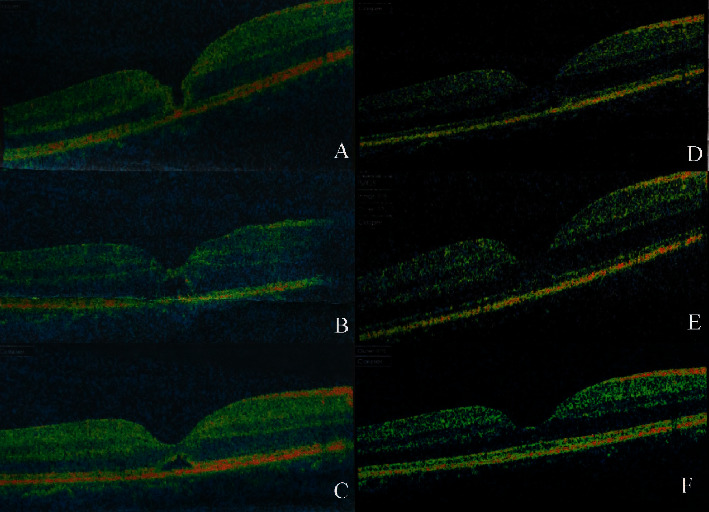
Case 3: a 14-year-old female patient with spontaneous macular hole closure. The patient sustained blunt trauma to the right eye from a firecracker. Her BCVA was 20/125 at initial and improved 120/200 after treatment. (a) OCT image 2 days after trauma showing FTMH. (b) OCT image 1 week, (c) 1 month, (d) 2 months, and (e) 3 months after trauma showed that a bridge connection has formed in the middle of the hole. (f) OCT image of 6 months after trauma, showing the neurosensory retina had almost connected, and the ellipsoid band had regained integrity.

**Figure 4:**
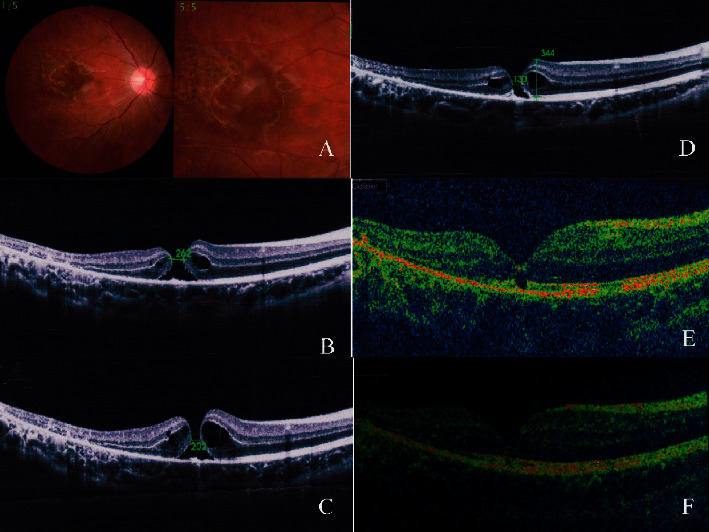
Case 5: a 12-year-old male patient with spontaneous macular hole closure. The patient sustained blunt trauma to the right eye from a racket. His BCVA was 20/63 at initial and improved 20/20 after treatment. (a) Color photo of the same eye 20 days after trauma showing retinal edema. (b) OCT image 20 days after trauma showing FTMH with narrowest diameter of 262 *μ*m. (c) OCT image 1 month after trauma; narrowest diameter decreased to 202 *μ*m. (d) OCT image 3 months after trauma; narrowest diameter decreased to 130 *μ*m. (e) OCT image 6 months after trauma; part of the ellipsoid band had not regained integrity. (f) OCT image 12 months after trauma; neurosensory retina and ellipsoid band had almost completely regained integrity.

A complete ellipsoid band could be seen in all OCT images of patients in the spontaneous closure group (Figures 3 and 4), while only 30.7% of patients (4/13) in the vitrectomy group retained the integrity of the ellipsoid band. A Fisher analysis confirmed that the integrity of the ellipsoid band was significantly correlated with visual acuity prognosis (Table 2).

### 3.4. Visual Outcome

**Figure 5:**
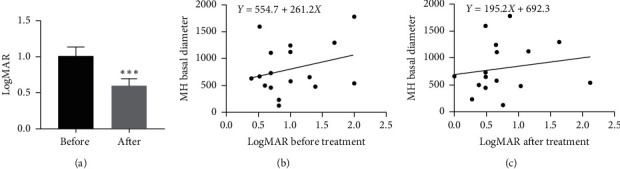
BCVA before and after treatment. (a) For all 17 eyes in our study, BCVA improved from median logMAR 1.01 ± 0.50 to 0.59 ± 0.40 at the final follow-up visit (*p* < 0.001). Preoperative macular hole basal diameter was positively correlated with (b) preoperative (*r* = 0.07, *p* = 0.2) and (C) postoperative (*r* = 0.02, *p* = 0.5) logMAR visual acuity.

For all 17 eyes in our study, the BCVA improved from logMAR 1.01 ± 0.50 to 0.59 ± 0.40 at the final follow-up visit (*p* < 0.001) (Figure 5(a)). In the 15 hole closure eyes, the BCVA improved from logMAR 0.98 ± 0.49 to 0.55 ± 0.39 (*p* = 0.01). In the four eyes with spontaneous hole closure, the BCVA improved from logMAR 0.86 ± 0.32 to 0.30 ± 0.25 (*p* = 0.03). In the 13 eyes with vitrectomy, the BCVA improved from logMAR 1.05 ± 0.54 to 0.68 ± 0.41 (*p* = 0.06). Preoperative basal diameter was positively correlated with preoperative (*r* = 0.07 and *p* = 0.2) and postoperative (*r* = 0.02 and *p* = 0.5) logMAR visual acuity (Figures 5(b) and 5(c)).

Complication: two gas-treated cases had a postoperative day 1 high intraocular pressure that was medically controlled for 1 week. None of the cases in the study developed serious complications, such as endophthalmitis, choroidal hemorrhage, or retinal detachment.

## 4. Discussion

Previously, very few studies reported the clinical and OCT features of TMH due to its low prevalence. Thus far, no clinical guidelines for TMH have been established. Therefore, it is necessary to explore the mechanism, examination, treatment, and prognosis of TMH.

### 4.1. Mechanism of TMH and Risk Factors

Many studies reported uncertain mechanism of TMH [3, 7, 9] with many hypotheses and speculations been put forward. The early-onset TMH is thought to be as a result of anteroposterior compression caused by blunt trauma, followed by a rebound contrecoup resulting in vitreofoveal traction [8]. During the rebound process, the tangential traction force between the vitreous and retina played an important role in the formation of the macular hole. We speculated that patients with preexisting posterior vitreous detachment (PVD) might have a lower risk of TMH formation. We all know that TMH patients are typically younger and have stronger vitreofoveal adhesion, which increases the risk of foveal avulsion. In China, a maximum sample size of 73 TMH eyes without PVD was recorded through careful clinical examination or by SD-OCT [13]. In a study of 20 patients with TMH, PVD was only found in three eyes (15%), and the vitreous was detached from the macula in only one eye (5%) [14]. In our study, only two of the 17 TMH eyes were found with PVD. The differentiation of PVD may be related to different methods of detecting PVD and examination time. Since PVD is generally detected during the patient evaluation, for some of these case reports, PVD could have been a result of injury rather than an antecedent event.

For delayed-onset TMH, ILM rupture and disruption in the retinal layers with secondary vitreous fluid accumulation may cause intraretinal cysts and swelling, which is also the reason for TMH formation [15]. Chen et al. [16] found that holes with spontaneous closure were less likely to have intraretinal cysts compared to holes that did not close spontaneously (10% versus 76.5%). They suggested that an absence of intraretinal cysts was the only independent predictive factor for spontaneous closure of TMH. Similar results were found in this study, with intraretinal cysts found in one of the four patients (25%) in the spontaneous closure group and in four of the thirteen patients (30.8%) in the vitrectomy group. However, there was not a statistically significant difference between the two groups.

### 4.2. Spontaneous Closure of TMH

In this study, we found that four eyes (23.5%) underwent spontaneous closure of TMH and the mean time of hole closure was 9.5 ± 9.9 weeks (range: 2–24 weeks), which is consistent with the literature report of 10–50% [7, 9, 16–18]. We also observed that the diameters of the holes in the spontaneous closure group were smaller than those in the vitrectomy group. Yamashita et al. [8] reported spontaneous closure in eight out of 18 (44.4%) TMH cases after a mean follow-up of 8.4 months. Li et al. [19] reported spontaneous closure in three out of 28 (10.7%) TMH cases after a mean follow-up of 18 months. Faghihi [7] reported six cases of spontaneous closure after 1 to 6 months of follow-up. Chen et al. [16] reported a 37.0% rate of spontaneous closure and found that those macular holes that closed spontaneously had a small minimum diameter and few intraretinal cysts. Therefore, Miller et al. [2] advised a 2- to 3-month observation period after TMH presentation. Although spontaneous hole closure can occur after another 3 months, this is less likely. Furthermore, extended observation beyond 3 months can lead to diminishing returns on successful hole closure via vitrectomy. Therefore, the experience of spontaneous closure of TMH suggests that a period of observation before surgical intervention may be recommended for the management of TMH. Based on our data, observation for 3 months may be considered before deciding if surgical intervention is suitable.

The mechanism of spontaneous closure of TMH is not clearly known. Lewis et al. [20] outlined that formation of the epiretinal membrane results in constriction of the hole. Takahashi and Kishi [21] suggested that reattachment of the operculum to the hole edge and bridging of the protruding retinal tissue over the hole can result in closure of the hole. On the contrary, Imai et al. [22] and Yamada et al. [9] observed no clear operculum during follow-up OCT and they supported that proliferation of glial cells and RPE cells from the edges of holes results in the filling of holes. Ishida et al. [23] noted that a complete detachment of the posterior hyaloid leads to a reduction in anteroposterior tractional forces and thus closure of the hole.

Our study identified two factors associated with spontaneous closure of TMH. The first factor was the small basal diameter of the macular hole at baseline, which may allow for easy migration of glial cells. The other associated factor was the integrity of the ellipsoid in the retina. In this study, OCT revealed that the ellipsoid band was intact in all patients with spontaneous closure of TMH, yet only four of the 13 eyes in the operation group had an intact ellipsoid band. The ellipsoid band is located in the photoreceptor layer of the retina, which is closely related to the RPE cell layer. During the repair process, glial cells and RPE at the edge of the hole proliferate and repair the hole, alleviating the edema of the photoreceptor layer and restoring the ellipsoid band (Figures 3 and 4).

### 4.3. Ellipsoid Band and Basal Diameter of OCT

OCT plays an important role in the follow-up of TMH to delineate the anatomic details of the defect, determine associated retinal pathology, and assess its progression objectively on a weekly or monthly basis. Our study revealed that the visual acuity was closely related to the integrity of the ellipsoid band. Miller [18] analyzed macular morphology on the OCT of 13 TMH eyes and concluded that there was a positive trend of better final acuity in both surgically managed eyes and those with spontaneous closure with the presence of an intact ellipsoid band. OCT showed that basal diameter was also a factor for TMH prognosis. Our study also confirmed that preoperative basal diameter was positively correlated with preoperative and postoperative logMAR visual acuity. Additionally, in our study, the preoperative BCVA of three eyes was very poor, and postoperative visual did not have obvious improvement which accompany on serious complications, including choroid rupture and subretinal hemorrhage.

OCT also allows for evaluation of the changes in the macular hole and guides surgical intervention. In the follow-up of cases with an aggravating tendency, it was observed through OCT that the basal diameter of holes increased in size from presentation to the last follow-up, which indicates a poor prognosis and merits further surgical intervention. Our results reported that the median time from clinical presentation to the operation was 2.3 ± 0.8 months. Therefore, an OCT observation period of 2–3 months after trauma was recommended.

### 4.4. Hole Closure of TMH

At present, the surgical treatment of choice for TMH is controversial. Kelly and Wendel [24] and Wendel et al. [25] reported on the surgical treatment of IMH for the first time. Most of the studies revealed that vitrectomy with or without ILM peeling remains an effective method for closing holes and improving vision for TMH [1, 26]. Our results showed that vitrectomy combined with ILM peeling is an optimal choice and the closure rate of holes after vitrectomy was 84.6%. A meta-analysis of surgical outcomes in all published reports of vitrectomy for TMH found a successful closure rate of 83% [1]. The multicenter trial by Johnson et al. [26] found successful closure in 24 of 25 cases (96%) of TMH undergoing vitrectomy. The reason for this difference in the hole closure rate may be the different criteria for evaluating hole closure. Kuhn et al. [27] defined macular hole closure as the disappearance of subretinal fluid and flattening of the hole edges. Ghoraba et al. [28] defined W-pattern closure as when the edges of the macular hole flatten against the RPE, though with a persistent full-thickness defect in the neurosensory retina. However, in our study, we defined not closure as a persistent full-thickness defect in the neurosensory retina, though the edge of the macular hole is attached to the RPE, which is the same as a W-pattern closure. Therefore, the percentage of holes closed was lower compared to other studies.

**Figure 6:**
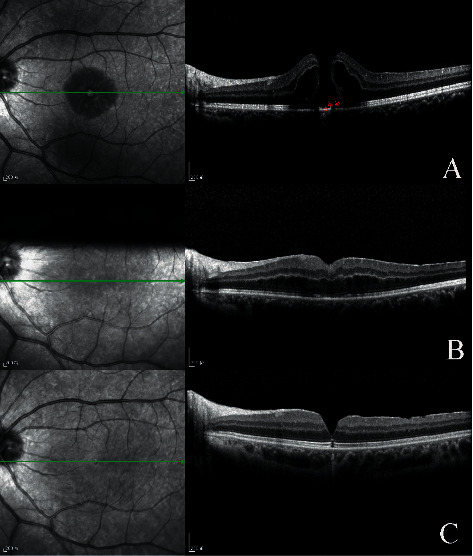
Case 11: a 45-year-old male patient with macular holes closed by vitrectomy combined with ILM peeling and C3F8 tamponade. The patient's left eye was damaged by a laser. His BCVA was 20/200. at initial and improved 20/63 after surgery. (a) Preoperative OCT image showing FTMH with intraretinal cyst. (b) OCT image 2 weeks postoperative showing the closed hole and intraretinal edema. (c) OCT image 4 months postoperative showing closed hole, but ellipsoid band had not regained integrity. His BCVA was now 20/63.

**Figure 7:**
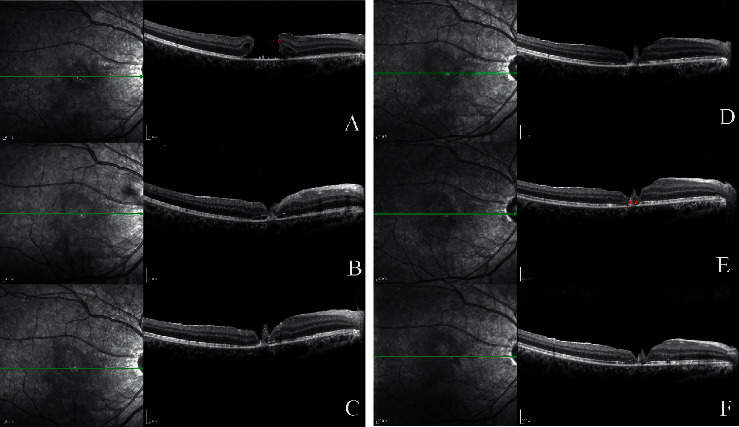
Case 15: a 16-year-old male patient with macular holes closed by vitrectomy. The patient sustained blunt trauma to the right eye from a fist. His BCVA was 20/500 at initial and improved 20/100 after surgery. (a) Preoperative OCT image showing FTMH with basal diameter of 1781 *μ*m. (b) OCT image 2 weeks after vitrectomy combined with ILM peeling and ILM flap and air tamponade. Retinal connection is visible, but part of the neuroretina and ellipsoid band has not regained integrity. OCT image (c) 1 month, (d) 4 months, (e) 9 months, and (f) 12 months postoperative.

Another reason for the differences in closure rates could be surgery management in terms of whether or not adjunctive is used during the operation. Macular hole closure via vitrectomy involves the stimulation of glial cell proliferation in the hole [29]. TGF-beta 2, platelet concentrate, and serum have been described as surgical adjuvants for the closure of IMH [24, 25]. These adjuvants can also be used in the repair of TMH. In our study, the standard surgical procedure was vitrectomy, followed by ILM peeling combined with gas tamponade, and then ILM flap and serum tamponade. We speculated that these adjuvants and the ILM flap may act as a scaffold, which could induce proliferation and migration of glial cells and assist in the formation of chorioretinal adhesion, thus enabling macular hole closure (Figures 6 and 7).

### 4.5. Treatment of TMH: Silicone Oil versus Gas

Silicone oil has also been used for TMH closure in some cases. Brasil and Brasil [30] reported a case of a nine-year-old boy with a TMH who was treated with ILM peeling and silicone oil tamponade and who gained better vision. In our study, none of the patients were treated with silicone oil. We believe that the gas is sufficient to facilitate the closure of the macular hole. The surface tension of silicone oil is lower than that of gas, and silicone oil will not increase the success rate of hole closure. In addition, silicone oil can cause burdens for the patient, such as the need for a second operation, increased risk of cataract, high intraocular pressure, and other surgical complications.

### 4.6. Limitations

This study had several limitations. Firstly, its retrospective design inevitably carries biases inherent in such studies. For example, the follow-up schedule was not consistent, and OCT scan was not performed on a regular basis. Secondly, the sample size was limited, and the results may not be generalizable to all populations. Thirdly, the most important limitation may be the method of OCT image acquisition. Different OCT instrument models were used for the same patient, and the image quality was variable. This may have affected the accuracy of measurement of ellipsoid band attenuation. Lastly, we did not have a comparative study on adjuvant serum use in vitrectomy management, which is mainly due to the low incidence of TMH. Therefore, the use of serum to promote the closure of TMH may lead to biased conclusions.

## 5. Conclusion

In conclusion, our study showed there is a moderate rate (23.5%) of spontaneous closure after the occurrence of TMH, while vitrectomy can result in an even better closure rate (84.6%). The mean time for the spontaneous closure of the hole was 9.5 ± 9.9 weeks, and 75% of the hole closure occurred within three months. Intraretinal cystic edema on the edge of the hole may be an unfavorable factor for the spontaneous closure of TMH. The basal diameter was smaller in the spontaneous closure group than that in the vitrectomy group. The ellipsoid band integrity was found to be closely correlated with visual acuity. Greater ellipsoid band attenuation was found in eyes with worse postoperative visual acuity. In all TMH patients, BCVA after treatment was significantly improved compared with that occurrence of TMH. Preoperative basal diameter was positively correlated with preoperative and postoperative logMAR visual acuity, yet there are no significant differences. In treatment, vitrectomy is an effective treatment for TMH, and the gas is sufficient to facilitate the closure of the macular hole.

## Figures and Tables

**Table 1:** All patient's details, surgery, and outcome.

Case	Age/gender	Eye	Type of trauma	Initial visual acuity	Macular hole size	Final visual acuity	Time from trauma to PPV	Surgical details	Adjunct	Outcome and complication
1	24/M	Right	Laser	20/63	1057	20/50	6 m	PPV + ILM peeling + C3F8	—	Closed
2	38/F	Right	Firecracker	20/200	1129	20/160	1 m	PPV + ILM peeling + C3F8	—	Closed
3	14/F	Right	Firecracker	20/125	231	20/32	—	—	—	Closed spontaneously
4	45/M	Right	Firecracker	20/100	1111	20/63	7 m	PPV + ILM peeling + C3F8	—	Not closed
5	12/M	Right	Racket	20/63	663	20/20	—	—	—	Closed spontaneously
6	19/M	Left	Bump	20/800	1298	20/50	3 m	PPV + ILM peeling + C2F6	—	Not closed (choroid rapture)
7	12/M	Left	Racket	20/80	498	20/40	2 m	PPV + ILM peeling + air	—	Closed
8	40/M	Right	Belt	20/100	732	20/400	5 m	PPV + ILM peeling + air		Closed
9	12/M	Left	Blunt	20/400	658	20/50	—	—	—	Closed spontaneously
10	10/M	Right	Racket	20/125	120	20/80	—	—	—	Closed spontaneously
11	45/M	Left	Laser	20/200	1242	20/63	2 w	PPV + ILM peeling + C3F8	ILM flap + serum	Closed
12	40/M	Right	Firecracker	20/80	451	20/50	3 w	PPV + ILM peeling + *C*3F8		Closed
13	17/M	Left	Football	20/500	475	20/125	1 m	PPV + ILM peeling + C3F8	—	Closed (subretinal hemorrage)
14	12/M	Right	Racket	20/80	627	20/50	3 m	PPV + ILM peeling + air	—	Closed
15	16/M	Right	Fist	20/500	1781	20/100	1 m	PPV + ILM peeling + air	ILM flap + serum	Closed
16	26/M	Right	Fist	20/500	540	20/500	3 w	PPV + ILM peeling + C3F8	ILM flap + serum	Closed (hemorrage; choroid rapture; orbit fracture)
17	17/M	Right	Racket	20/200	579	20/63	1 m	PPV + ILM peeling + C3F8	—	Closed

**Table 2:** The relationship between ellipsoid zone and visual acuity prognosis.

Ellipsoid band zone	Spontaneous closure group	Vitrectomy group	Fisher's exact test
With	4	4	*p* = 0.029
Without	0	9	

## Data Availability

The data used to support the findings of this study are included within the supplementary information files.
